# Association of Intraindividual Difference in Estimated Glomerular Filtration Rate by Creatinine vs Cystatin C and End-stage Kidney Disease and Mortality

**DOI:** 10.1001/jamanetworkopen.2021.48940

**Published:** 2022-02-17

**Authors:** Debbie C. Chen, Michael G. Shlipak, Rebecca Scherzer, Scott R. Bauer, O. Alison Potok, Dena E. Rifkin, Joachim H. Ix, Anthony N. Muiru, Chi-yuan Hsu, Michelle M. Estrella

**Affiliations:** 1 Division of Nephrology, Department of Medicine, University of California, San Francisco; 2 Kidney Health Research Collaborative, San Francisco VA Health Care System & University of California, San Francisco; 3 Department of Medicine, San Francisco VA Medical Center, San Francisco, California; 4 Department Epidemiology and Biostatistics, University of California, San Francisco; 5 Division of General Internal Medicine, University of California, San Francisco; 6 Division of Nephrology and Hypertension, Department of Medicine, University of California, San Diego; 7 Nephrology Section, Veterans Affairs San Diego Healthcare System, San Diego, California; 8 Division of Nephrology, Department of Medicine, San Francisco VA Medical Center, San Francisco, California

## Abstract

**Importance:**

As cystatin C is increasingly adopted to estimate glomerular filtration rate (eGFR), clinicians will encounter patients in whom cystatin C-based eGFR (eGFRcys) and creatinine-based eGFR (eGFRcr) differ widely. The clinical implications of these differences, eGFRdiff_cys-cr_, are unknown.

**Objective:**

To evaluate the associations of eGFRdiff_cys-cr_ with end-stage kidney disease (ESKD) and mortality among individuals with chronic kidney disease (CKD).

**Design, Setting, and Participants:**

This is a prospective cohort study of 4956 individuals with mild to moderate CKD from 7 clinical centers in the United States who enrolled in the Chronic Renal Insufficiency Cohort (CRIC) Study between 2003 to 2018. Statistical analyses were completed in December 2021.

**Exposures:**

eGFRdiff_cys-cr_ (eGFRcys − eGFRcr) was calculated at baseline and annually thereafter for 3 years. Because 15 mL/min/1.73 m^2^ represents a clinically meaningful difference in eGFR that also distinguishes CKD stages, eGFRdiff_cys-cr_ was categorized as: less than −15 mL/min/1.73 m^2^, −15 to 15 mL/min/1.73 m^2^, and 15 mL/min/1.73 m^2^ or greater.

**Main Outcomes and Measures:**

The outcomes of ESKD, defined as initiation of maintenance dialysis or receipt of a kidney transplant, and all-cause mortality were adjudicated from study entry until administrative censoring in 2018.

**Results:**

Among 4956 participants with mean (SD) age of 59.5 (10.5) years, 2152 (43.4%) were Black, 515 (10.4%) were Hispanic, and 2113 (42.6%) were White. There were 2156 (43.5%) women and 2800 (56.5%) men. At baseline, eGFRcys and eGFRcr values differed by more than 15 mL/min/1.73 m^2^ in one-third of participants (1638 participants [33.1%]). Compared with participants with similar baseline eGFRcys and eGFRcr (eGFRdiff_cys-cr_ −15 to 15 mL/min/1.73 m^2^), those in whom eGFRcys was substantially lower than eGFRcr (eGFRdiff_cys-cr_ < −15 mL/min/1.73 m^2^) had a higher risk of mortality (hazard ratio [HR], 1.86; 95% CI, 1.40-2.48) while those with eGFRdiff_cys-cr_ of 15 mL/min/1.73 m^2^ or greater had lower risks of ESKD (subHR [SHR], 0.73; 95% CI, 0.59-0.89) and mortality (HR, 0.68; 95% CI, CI 0.58-0.81). In time-updated analyses, participants with eGFRdiff_cys-cr_ less than −15 mL/min/1.73 m^2^ had higher risks of ESKD (SHR, 1.83; 95% CI, 1.10-3.04) and mortality (HR, 3.03; 95% CI, 2.19-4.19) compared with participants with similar eGFRcys and eGFRcr. Conversely, participants with eGFRdiff_cys-cr_ of 15 mL/min/1.73 m^2^ or greater had lower risks of ESKD (SHR, 0.50; 95% CI, 0.35-0.71) and mortality (HR, 0.58; 95% CI, 0.45-0.75). Longitudinal changes in eGFRdiff_cys-cr_ were associated with mortality risk. Compared with participants who had similar slopes by eGFRcys and eGFRcr, those with smaller eGFRcr declines had an 8-fold increased mortality risk (HR, 8.20; 95% CI, 6.37-10.56), and those with larger apparent declines by eGFRcr had a lower mortality risk (HR, 0.14; 95% CI, 0.08-0.24).

**Conclusions and Relevance:**

These findings suggest that large differences between eGFRcys and eGFRcr were common in persons with CKD. These differences and their changes over time may be informative of ESKD and mortality risks, warranting monitoring of both eGFRcys and eGFRcr in this high-risk population.

## Key Points

**Question:**

Are differences between estimated glomerular filtration rate by cystatin C (eGFRcys) vs creatinine (eGFRcr) associated with the risk of end-stage kidney disease (ESKD) and mortality among individuals with chronic kidney disease (CKD)?

**Findings:**

In this cohort study of 4956 participants with CKD, differences between eGFRcys and eGFRcr identified high- and low-risk groups with more than 3-fold difference in ESKD risk and 5-fold difference in mortality risk. Divergence between eGFRcys and eGFRcr slopes over time was strongly associated with mortality risk.

**Meaning:**

These findings suggest that among patients with CKD, longitudinal monitoring of both cystatin C and creatinine may be warranted to enhance risk stratification.

## Teaser

This cohort study evaluates associations of differences in glomerular filtration rate estimated by cystatin C vs by creatinine with end-stage kidney disease and mortality.

## Introduction

The burden of chronic kidney disease (CKD) in the US is substantial, with more than 130 000 persons progressing to end-stage kidney disease (ESKD) each year.^1^ Estimated glomerular filtration rate (eGFR) is crucial for staging and risk-stratifying persons with CKD, as lower eGFR has an independent, graded association with higher risk for ESKD and mortality.^2,3,4^ Creatinine and cystatin C are biomarkers used to determine eGFR. However, cystatin C–based eGFR (eGFRcys) and creatinine-based eGFR (eGFRcr) measured in the same individual may be highly discrepant.^5,6^ Currently, there is no guidance for CKD risk stratification when eGFRcys and eGFRcr are substantially different, and the clinical implications of these differences are unclear.

Differences between eGFRcys and eGFRcr, defined as *eGFRdiff_cys-c_*_r_ = *eGFRcys* − *eGFRcr*, may occur when factors unrelated to kidney function differentially affect creatinine and cystatin C. The eGFRcr equation was derived to account for non-GFR factors associated with creatinine levels, including age and sex.^7^ However other non-GFR factors that are associated with creatinine levels, including physical activity, muscle mass, and diet, are unaccounted for and may lead to overestimation or underestimation of kidney function by eGFRcr.^8,9,10,11,12,13^ Recently, the National Kidney Foundation (NKF) and American Society of Nephrology (ASN) recommended the increased use of cystatin C to estimate GFR because cystatin C is less affected than creatinine by non-GFR determinants and is not associated with race or genetic ancestry.^7,14,15,16^ The NKF and ASN also recommended using the combined eGFR equation, which includes both cystatin C and creatinine to balance the potential confounding by their non-GFR determinants.^15^ However, a large eGFRdiff_cys-cr_ indicates that non-GFR factors are associated with substantial change in 1 biomarker; therefore, use of the combined eGFR equation among persons with large eGFRdiff_cys-cr_ may mask the differential influence of these non-GFR factors.

Current practice guidelines for CKD recommend measuring cystatin C when precision of eGFR is required and suggest adding cystatin C for confirmatory testing in situations when creatinine is less accurate, such as in persons who may underproduce creatinine because they are frail.^5,6,17^ As efforts advance to improve CKD detection and monitoring and to eliminate the use of race in estimating kidney function, cystatin C will be increasingly incorporated into routine clinical care, and clinicians will commonly encounter persons with large eGFRdiff_cys-cr_.^5,6,18,19^

Persons with large eGFRdiff_cys-cr_ may have different risk profiles from those with similar eGFRcys and eGFRcr values. Furthermore, the eGFRdiff_cys-cr_ would evolve if eGFRcys and eGFRcr changed at different rates over time, thus potentially offering further prognostic information. Two prior studies have demonstrated associations of baseline eGFRdiff_cys-cr_ with mortality risk,^5,6^ but the implications of changes in eGFRdiff_cys-cr_ are unknown. Furthermore, examining kidney-specific end points, such as ESKD, among a CKD population is important for determining the prognostic utility of eGFRcys as a component of eGFRdiff_cys-cr_. In this study, we sought to address the following questions among a cohort of individuals with CKD: (1) is eGFRdiff_cys-cr_ at baseline independently associated with progression to ESKD and mortality; (2) compared with baseline values, do time-updated measures of eGFRdiff_cys-cr_ yield different associations with ESKD and mortality; and (3) are longitudinal divergences between eGFRcys and eGFRcr independently associated with progression to ESKD and mortality?

## Methods

This cohort study protocol was approved by the institutional review boards at each participating site. All participants provided written informed consent. This study followed the Strengthening the Reporting of Observational Studies in Epidemiology (STROBE) reporting guideline.

### Study Design and Population

The Chronic Renal Insufficiency Cohort (CRIC) Study is a multicenter observational cohort study that enrolled 5499 adults from 7 clinical centers across the US.^20,21,22^ Participants at study entry were aged 21 to 74 years and had eGFRcr of 20 to 70 mL/min/1.73 m^2^. Medical history, medication use, and clinical events were updated semiannually. Laboratory testing was conducted annually. Additional details on the study design and population have been published elsewhere.^20,21,22^ In this study, all participants who had simultaneous serum cystatin C and creatinine measurements from at least 2 study visits were included. We excluded 543 participants who did not meet this inclusion criterion.

### Independent Variable

Our independent variable of interest was eGFRdiff_cys-cr_, defined as *eGFRcys* − *eGFRcr*. Serum cystatin C and creatinine were measured annually in the CRIC Study and applied to corresponding 2009 and 2021 CKD Epidemiology Collaboration (CKD-EPI) equations, respectively, to calculate eGFR.^7,23^ Additional details regarding cystatin C and creatinine assays are in the eMethods of the Supplement.

### Outcomes

Our outcomes were ESKD and all-cause mortality. ESKD was defined as initiation of maintenance dialysis or receipt of a kidney transplant and was ascertained through self-report or by report of the participant’s named contact, supplemented by the US Renal Data System.^1^ All-cause mortality was identified through report by family members, death certificates or obituaries, hospitalization records, and the Social Security Death Index.^20^ Outcomes were adjudicated from study entry until administrative censoring in 2018.

### Covariates

All covariates were obtained at the baseline study visit concurrently with serum cystatin C and creatinine. Demographic characteristics, medical history, and medication use were self-reported. Race and ethnicity were self-reported and included as a demographic characteristic for adjustment in our models. Blood and urine were collected according to study protocol at baseline and at annual study visits.^20^ Urine protein-to-creatinine ratio (UPCR) was obtained from 24-hour or spot urine samples. If both were available, the 24-hour UPCR was used. In our time-updated analyses, eGFRcr, UPCR, and waist circumference were time-updated covariates.

### Statistical Analysis

We summarized baseline characteristics in the analytic cohort overall and stratified by 3 eGFRdiff_cys-cr_ categories: lower than −15 mL/min/1.73 m^2^, with eGFRcys lower than eGFRcr; −15 to 15 mL/min/1.73 m^2^, with eGFRcys similar to eGFRcr; and 15 mL/min/1.73 m^2^ or greater, with eGFRcys higher than eGFRcr. These eGFRdiff_cys-cr_ cutoffs were chosen because 15 mL/min/1.73 m^2^ corresponds to approximately 1-SD of baseline eGFRdiff_cys-cr_, represents a clinically meaningful difference in eGFR that distinguishes CKD stages, and has been used in prior studies to categorize eGFRdiff_cys-cr_.^5,6,17^ Differences across baseline eGFRdiff_cys-cr_ categories were compared using χ^2^, analysis of variance, and Kruskal-Wallis tests.

To investigate the association between baseline eGFRdiff_cys-cr_ and ESKD, we applied Fine and Gray proportional subhazards regression, with death modeled as a competing risk.^24^ The primary exposure of eGFRdiff_cys-cr_ was analyzed as a categorical variable, compared with the reference group of eGFRdiff_cys-cr_ between −15 to 15 mL/min/1.73 m^2^. We initially adjusted for age, sex, race or ethnicity, and baseline eGFRcr. We adjusted for eGFRcr to assess the prognostic value of eGFRdiff_cys-cr_ independent of the most common measure of kidney function in current clinical practice. Our fully adjusted model included diabetes, hypertension, cardiovascular disease (CVD), heart failure, amputation, chronic obstructive pulmonary disease (COPD), angiotensin converting-enzyme inhibitor or angiotensin receptor blocker use, steroid use, UPCR, and waist circumference. In exploratory analyses, we additionally adjusted for serum albumin, hemoglobin, and C-reactive protein (CRP) concentrations to determine whether these markers of health status would attenuate the associations of eGFRdiff_cys-cr_ with the 2 end points. We log-transformed UPCR and CRP because of their right-skewed distributions. To assess the association between baseline eGFRdiff_cys-cr_ and all-cause mortality, we used multivariable Cox proportional hazards regression analyses, adjusting for the same baseline covariates as the ESKD models.

Next, we modeled eGFRdiff_cys-cr_, eGFRcr, UPCR, and waist circumference as time-updated variables, and we repeated the Fine and Gray and Cox regression analyses for the outcomes of ESKD and mortality, respectively. In addition to time-updated measures of kidney function, we adjusted for time-updated waist circumference because weight loss becomes increasingly prevalent as CKD progresses and represents an important potential confounder.^25^

Longitudinal changes in eGFRdiff_cys-cr_ can be interpreted as the relative divergence of the eGFRcr slope from the eGFRcys slope. We used joint models to obtain within-participant estimates of eGFRdiff_cys-cr_ intercept and slope.^26,27,28^ Additional details regarding our joint models are included in the eMethods in the Supplement. We created tertiles of eGFRdiff_cys-cr_ slope. In the first tertile, the most negative eGFRdiff_cys-cr_ slope, eGFRcr does not decline as quickly as eGFRcys. In the third tertile, the most positive eGFRdiff_cys-cr_ slope, eGFRcr declines faster than eGFRcys. We evaluated these tertiles as independent variables associated with ESKD and all-cause mortality in time-to-event models.

We conducted several secondary analyses to further explore the association of eGFRdiff_cys-cr_ with outcomes. We tested whether associations between eGFRdiff_cys-cr_ and outcomes differed by a set of baseline characteristics through stratified analyses and inclusion of interaction terms. The a priori selected baseline characteristics included age younger than 60 years vs 60 years or older, female or male sex, self-identified Black or non-Black race (including Hispanic, non-Hispanic White, and other race or ethnicity, including American Indian or Alaska Native, Asian, Native Hawaiian or other Pacific Islander, >1 race or ethnicity, or unknown), and eGFRcr less than 45 mL/min/1.73 m^2^ vs 45 mL/min/1.73 m^2^ or greater. We also used joint models to obtain eGFRcys and eGFRcr slope estimates and determined their individual associations with ESKD using a Fine-Gray model and mortality using a Cox model.

Methods regarding model diagnostics and handing of missing data are included in the eMethods in the Supplement. All tests were 2-tailed with a statistical significance level of *P* < .05. Statistical analyses were performed using SAS version 9.4 (SAS Institute) and R version 4.1.0 (R Project for Statistical Computing). Statistical analyses were completed in December 2021.

## Results

Among 4956 CRIC Study participants, 2156 (43.5%) were women and 2800 (56.5%) men, and the mean (SD) age was 59.5 (10.5) years. A total of 2152 participants (43.4%) were non-Hispanic Black, 515 (10.4%) were Hispanic, and 2113 (42.6%) were White. At baseline, mean (SD) eGFRcys was 54 (23) mL/min/1.73 m^2^, and mean (SD) eGFRcr was 49 (16) mL/min/1.73 m^2^ (Table 1). Baseline eGFRdiff_cys-cr_ ranged from −52 to 65 mL/min/1.73 m^2^, with a mean (SD) of 6 (16) mL/min/1.73 m^2^ (eFigure 1 in the Supplement). Approximately two-thirds of participants had a baseline eGFRdiff_cys-cr_ between −15 and 15 mL/min/1.73 m^2^ (3318 participants [66.9%]; midrange eGFRdiff_cys-cr_); 390 participants (7.9%) had an eGFRdiff_cys-cr_ less than −15 mL/min/1.73 m^2^ (negative eGFRdiff_cys-cr_), and 1248 participants (25.2%) had an eGFRdiff_cys-cr_ of 15 mL/min/1.73 m^2^ or greater (positive eGFRdiff_cys-cr_). Discrepancies between eGFRcys and eGFRcr were smallest among those with eGFRcr less than 30 mL/min/1.73 m^2^ and widened at higher eGFRcr values (eFigure 2 in the Supplement). Compared with the other 2 eGFRdiff_cys-cr_ groups, participants in the negative eGFRdiff_cys-cr_ group were generally older and had the highest prevalence of baseline comorbidities, including diabetes, CVD, and heart failure (Table 1).

**Table 1.** Baseline Characteristics of Participants by Category of Baseline eGFRdiff_cys-cr_ in the Chronic Renal Insufficiency Cohort Study

Characteristic	Participants, No. (%)				
	Overall (N = 4956)	Baseline eGFRdiff_cys-cr_ category, mL/min/1.73 m^2^			
		<−15 (Negative) (n = 390)	−15 to 15 (Midrange) (N = 3318)	≥15 (Positive) (n = 1248)	*P* value
Age, mean (SD), y	59.5 (10.5)	63.0 (9.1)	59.9 (10.6)	57.5 (10.6)	<.001
Sex					
Men	2800 (56.5)	243 (62.3)	1855 (55.9)	702 (56.3)	.05
Women	2156 (43.5)	147 (37.7)	1463 (44.1)	546 (43.8)	
Race and ethnicity					
Black, Non-Hispanic	2152 (43.4)	133 (34.1)	1369 (41.3)	650 (52.1)	<.001
Hispanic	515 (10.4)	35 (9.0)	424 (12.8)	56 (4.5)	
White, Non-Hispanic	2113 (42.6)	207 (53.1)	1407 (42.4)	499 (40.0)	
Other^a^	176 (3.6)	15 (3.8)	118 (3.6)	43 (3.4)	
Comorbidities					
Diabetes	2495 (50.3)	235 (60.3)	1808 (54.5)	452 (36.2)	<.001
Hypertension	4270 (86.2)	339 (87.1)	2981 (89.8)	950 (76.1)	<.001
Cardiovascular disease	1625 (32.8)	156 (40.0)	1185 (35.7)	284 (22.8)	<.001
Heart failure	445 (9.0)	50 (12.8)	342 (10.3)	53 (4.2)	<.001
History of stroke	503 (10.1)	52 (13.3)	353 (10.6)	98 (7.9)	.002
COPD	263 (5.4)	45 (11.7)	178 (5.5)	40 (3.2)	<.001
Amputation	143 (4.0)	15 (16.3)	114 (4.5)	14 (1.4)	<.001
Waist circumference, mean (SD), cm	106.9 (17.5)	113.6 (19.4)	108.0 (17.8)	102.0 (14.9)	<.001
Medications					
ACEI or ARB	3388 (68.8)	274 (70.4)	2372 (72.0)	742 (59.7)	<.001
Steroids	615 (12.5)	77 (19.8)	404 (12.3)	134 (10.8)	<.001
Laboratory values					
eGFRcys, mean (SD), mL/min/1.73 m^2^	54 (23)	37 (13)	46 (16)	81 (18)	<.001
eGFRcr, mean (SD), mL/min/1.73 m^2^	49 (16)	61 (13)	45 (15)	55 (13)	<.001
UPCR, median (IQR), g/g	0.14 (0.06-0.64)	0.21 (0.08-0.78)	0.20 (0.07-0.88)	0.07 (0.04-0.17)	<.001
Albumin, mean (SD), g/dL	4.0 (0.5)	3.9 (0.4)	3.9 (0.5)	4.1 (0.4)	<.001
Hemoglobin, mean (SD), g/dL	12.7 (1.8)	12.6 (1.9)	12.6 (1.8)	13.2 (1.6)	<.001
High-sensitivity CRP, median (IQR), mg/L	2.5 (1.0-6.3)	4.3 (1.4-8.8)	2.8 (1.1-6.9)	1.7 (0.9-4.2)	<.001

Abbreviations: ACEI, angiotensin-converting enzyme; ARB, angiotensin-receptor blocker; COPD, chronic obstructive pulmonary disease; CRP, C-reactive protein; eGFRcr, creatinine-based estimated glomerular filtration rate using the 2021 Chronic Kidney Disease Epidemiology Collaboration equation; eGFRcys, cystatin C-based eGFR; eGFRdiff_cys-cr_, difference between eGFRcys and eGFRcr; UPCR, urine protein-to-creatinine ratio.

SI conversion factors: To convert albumin to grams per liter, multiply by 10; hemoglobin to grams per liter, multiply by 10.

^a^ Other race or ethnicity included American Indian or Alaska Native, Asian, Native Hawaiian or other Pacific Islander, more than 1 race, or unknown.

### Association of Baseline eGFRdiff_cys-cr_ With ESKD

**Figure 1.**
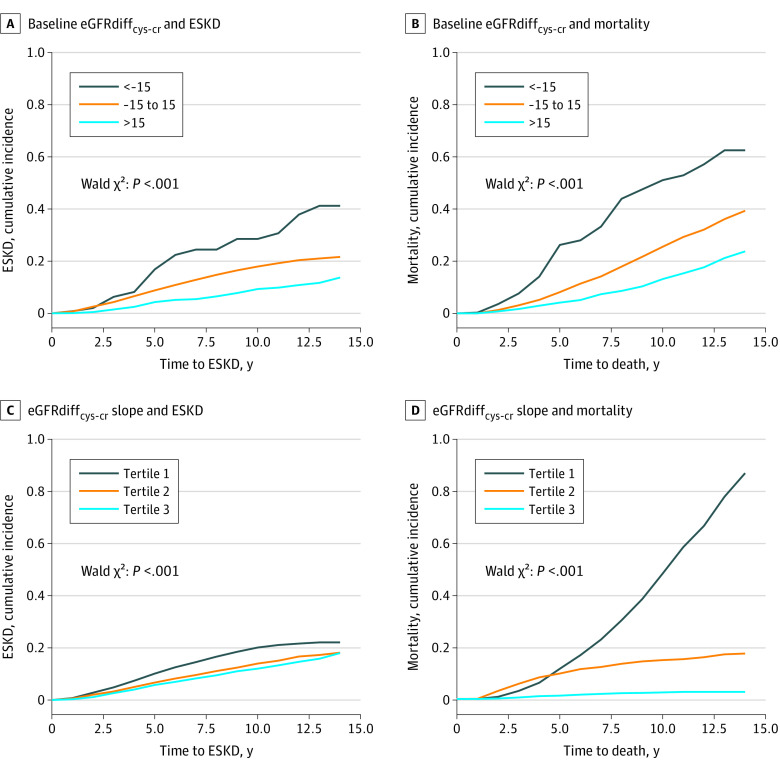
Demographically Adjusted Cumulative Incidence of End-stage Kidney Disease (ESKD) and All-Cause Mortality by Baseline Difference in Cystatin C– and Creatinine-Based Estimated Glomerular Filtration Rate (eGFRdiff_cys-cr_)

A total of 1173 participants (23.7%) developed ESKD, with median (IQR) time until ESKD of 4.7 (2.6-7.5) years. After adjusting for demographic characteristics and eGFRcr, participants in the positive eGFRdiff_cys-cr_ group had a lower risk of ESKD compared with the midrange eGFRdiff_cys-cr_ group (subhazard ratio [SHR], 0.51; 95% CI, 0.42-0.62) (Table 2 and Figure 1A). Further multivariable adjustment using baseline covariates attenuated this association (SHR, 0.73; 95% CI, 0.59-0.89), but it remained statistically significant. The negative eGFRdiff_cys-cr_ category was not associated with risk of ESKD.

**Table 2.** Associations of eGFRdiff_cys-cr_ With End-stage Kidney Disease Risk, Using Fine and Gray Proportional Subhazards Models With Mortality as Competing Risk

Measure	Demographic-adjusted^a^		Fully adjusted^b^	
	Subhazard ratio (95% CI)	*P* value	Subhazard ratio (95% CI)	*P* value
**Baseline measures**				
Categorical eGFRdiff_cys-cr,_ mL/min/1.73 m^2^				
< −15	1.61 (1.14-2.26)	.006	1.00 (0.65-1.52)	.99
−15 to 15	1 [Reference]	NA	1 [Reference]	NA
≥15	0.51 (0.42-0.62)	<.001	0.73 (0.59-0.89)	.002
<−15 vs ≥15	3.15 (2.18-4.56)	<.001	1.37 (0.87-2.16)	.17
**Time-updated measures^c^**				
Categorical eGFRdiff_cys-cr_, mL/min/1.73 m^2^				
<−15	2.06 (1.18-3.58)	.01	1.83 (1.10-3.04)	.02
−15 to 15	1 [Reference]	NA	1 [Reference]	NA
≥15	0.39 (0.27-0.58)	<.001	0.50 (0.35-0.71)	<.001
<−15 vs ≥15	5.25 (2.78-9.94)	<.001	3.70 (2.05-6.65)	<.001
Slope of eGFRdiff**_cys-_**_cr_, mL/min/1.73 m^2^/y^d^				
Tertile 1, −7.9 to <−0.8	1.26 (1.05-1.52)	.01	1.19 (0.98-1.46)	.08
Tertile 2, −0.8 to −0.06	1 [Reference]	NA	1 [Reference]	NA
Tertile 3, >−0.06 to 24.2	0.78 (0.63-0.95)	.01	0.86 (0.70-1.06)	.16
Tertile 1 vs tertile 3	1.63 (1.31-2.02)	<.001	1.39 (1.12-1.71)	.002

Abbreviations: eGFRdiff_cys-cr_, difference between cystatin C– and creatinine-based estimated glomerular filtration rate; ESKD, end-stage kidney disease; NA, not applicable.

^a^ Demographic-adjusted model: adjusted for age, sex, race or ethnicity, and creatinine-based eGFR.

^b^ Fully adjusted model: adjusted for demographic-adjusted model and diabetes, hypertension, cardiovascular disease, heart failure, amputation, chronic obstructive pulmonary disease, angiotensin-converting enzyme or angiotensin-receptor blocker, steroids, log urine protein-to-creatinine ratio, and waist circumference.

^c^ All covariates are from the baseline examination except creatinine-based eGFR, urine protein-to-creatinine ratio, waist circumference, and eGFRdiff_cys-cr_, which were time-updated.

^d^ Within-participant slopes were estimated from a joint model of eGFR trajectory and survival or dropout. In slope models, all covariates were from baseline examination except creatinine-based eGFR, urine protein-to-creatinine ratio, and waist circumference, which were time-updated.

### Association of Time-Updated eGFRdiff_cys-cr_ With ESKD

In our fully adjusted model incorporating time-updated variables, participants within the positive eGFRdiff_cys-cr_ category had lower risk for ESKD compared with participants with midrange eGFRdiff_cys-cr_ (SHR, 0.50; 95% CI, 0.35-0.71); whereas participants in the negative eGFRdiff_cys-cr_ group had a higher risk of ESKD (SHR, 1.83; 95% CI, 1.10-3.04) (Table 2). This elevated risk was amplified to more than 3-fold when comparing the negative eGFRdiff_cys-cr_ group to the positive eGFRdiff_cys-cr_ group (SHR, 3.70; 95% CI, 2.05-6.65). These results remained robust after additional adjustment for markers of nutritional status and inflammation (eTable 1 in the Supplement).

### Association of eGFRdiff_cys-cr_ Slope With ESKD

**Figure 2.**
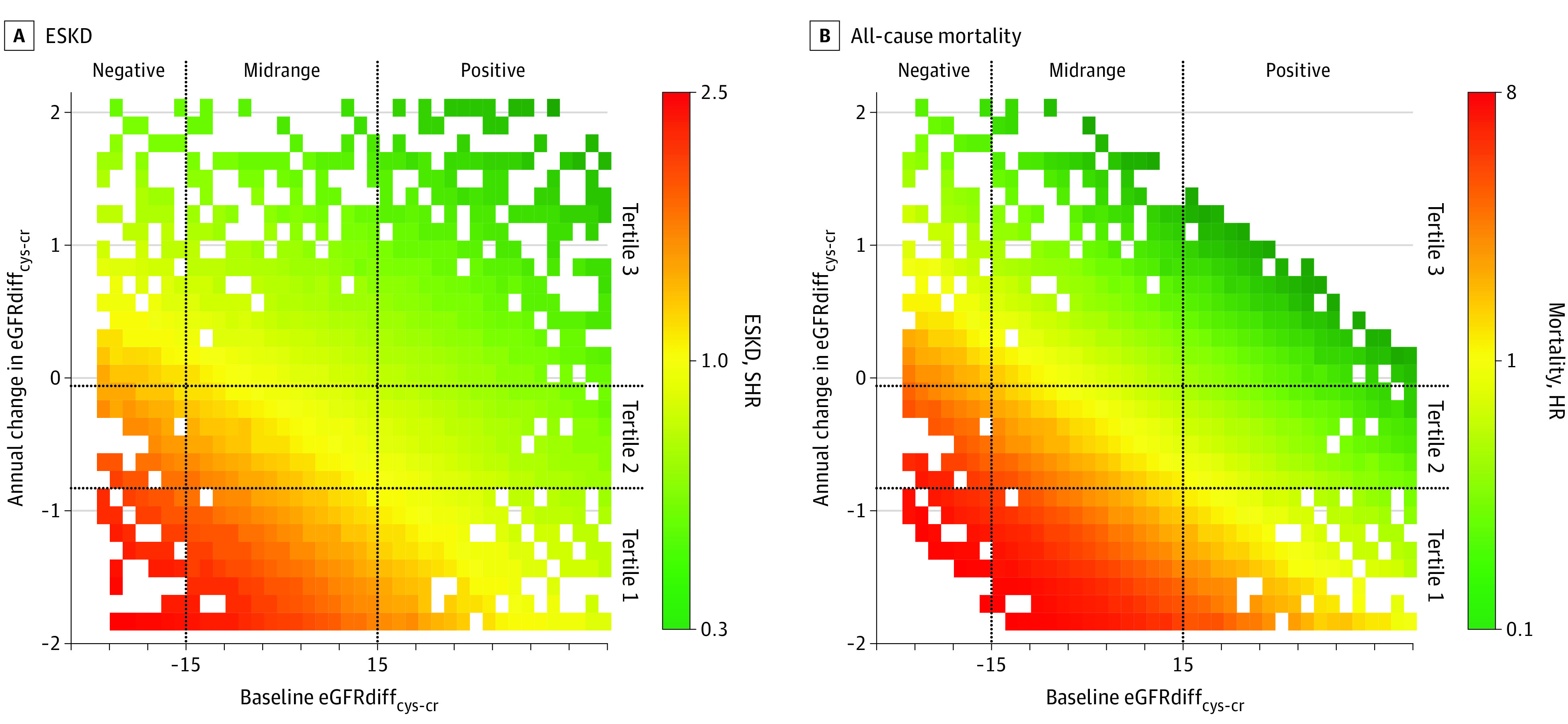
Heatmap of Unadjusted Associations of Baseline Difference in Creatinine- and Cystatin C–Based Estimated Glomerular Filtration Rate (eGFRdiff_cys-cr_) ESKD indicates end-stage kidney disease; HR, hazard ratio; and SHR, subhazard ratio.

Slopes of eGFRdiff_cys-cr_ were derived using a median (IQR) of 4 (3-4) eGFRdiff_cys-cr_ values, each obtained 1 year apart. The mean (SD) annual change in eGFRdiff_cys-cr_ was −0.4 (1.1) mL/min/1.73 m^2^ per year. Participants within the first tertile of eGFRdiff_cys-cr_ slope, among whom eGFRcys declined more steeply than eGFRcr, had a higher risk of ESKD than participants in the middle tertile with similar longitudinal changes in eGFRcys and eGFRcr in the demographics-adjusted model (SHR, 1.26; 95% CI, 1.05-1.52), but the difference was no longer significant in the fully adjusted model (SHR, 1.19; 95% CI, 0.98-1.46). Participants in the third tertile, with faster declines in eGFRcr than eGFRcys, had a lower risk of ESKD in the demographics-adjusted model (SHR, 0.78; 95% CI, 0.63-0.95), although again, these findings were not statistically significant in the fully adjusted model (SHR, 0.86; 95% CI, 0.70-1.06) (Table 2 and Figure 1A). As shown in Figure 2A, at all baseline eGFRdiff_cys-cr_ values, the risk for ESKD appeared higher for participants with declining eGFRdiff_cys-cr_.

### Association of eGFRdiff_cys-cr_ With All-cause Mortality

Death occurred in 1298 participants (26.2%), with a median (IQR) time until death of 7.2 (4.4-9.7) years. In fully adjusted models, participants in the negative eGFRdiff_cys-cr_ group had higher risk of mortality compared with the midrange eGFRdiff_cys-cr_ group in both the baseline (HR, 1.86; 95% CI, 1.40-2.48) and time-updated analyses (HR, 3.03; 95% CI, 2.19-4.19) (Table 3). Conversely, participants in the positive eGFRdiff_cys-cr_ group had lower risks for mortality in baseline (HR, 0.68; 95% CI, 0.58-0.81) and time-updated analyses (HR, 0.58; 95% CI, 0.45-0.75). In longitudinal slope analyses, 1020 of 1652 participants (61.7%) with eGFRcr declining more slowly than eGFRcys (first tertile of eGFRdiff_cys-cr_ slope) died; 452 of these deaths (44.3%) occurred after ESKD. In contrast, 33 of 1652 participants (2.0%) with faster declines in eGFRcr than eGFRcys (third tertile of eGFRdiff_cys-cr_ slope) died; 4 of these deaths (12.1%) occurred after ESKD (eTable 2 in the Supplement). Compared with participants with stable eGFRdiff_cys-cr_, those in the first tertile of eGFRdiff_cys-cr_ slope had an 8.2-fold greater adjusted risk of mortality (HR, 8.20; 95% CI, 6.37-10.56) and those in the third tertile of eGFRdiff_cys-cr_ slope had a lower risk of mortality (HR, 0.14; 95% CI, 0.08-0.24) (Table 3 and Figure 1D). These results were unaffected by adjustment for markers of nutritional status and inflammation (eTable 1 in the Supplement).

**Table 3.** Associations of eGFRdiff_cys-cr_ With All-Cause Mortality Using Cox Proportional Hazards Models

Measure	Demographic-adjusted^a^		Fully adjusted^b^	
	Hazard ratio (95% CI)	*P* value	Hazard ratio (95% CI)	*P* value
**Baseline measures**				
Categorical eGFRdiff_cys-cr,_ mL/min/1.73 m^2^				
< −15	2.40 (1.82-3.18)	<.001	1.86 (1.40-2.48)	<.001
−15 to 15	1 [Reference]	NA	1 [Reference]	NA
≥15	0.51 (0.44-0.60)	<.001	0.68 (0.58-0.81)	<.001
<−15 vs ≥15	4.69 (3.47-6.32)	<.001	2.73 (1.99-3.73)	<.001
**Time-updated measures^c^**				
Categorical eGFRdiff_cys-cr_, mL/min/1.73 m^2^				
< −15	3.59 (2.61-4.94)	<.001	3.03 (2.19-4.19)	<.001
−15 to 15	1 [Reference]	NA	1 [Reference]	NA
≥15	0.48 (0.37-0.61)	<.0001	0.58 (0.45-0.75)	<.001
<−15 vs ≥15	7.54 (5.19-10.96)	<.001	5.20 (3.54-7.64)	<.001
Slope of eGFRdiff_cys-cr_, mL/min/1.73 m^2^/y^d^				
Tertile 1, −7.9 to <−0.8	9.21 (7.17-11.83)	<.001	8.20 (6.37-10.56)	<.001
Tertile 2, −0.8 to −0.06	1 [Reference]	NA	1 [Reference]	NA
Tertile 3, >−0.06 to 24.2	0.15 (0.09-0.26)	<.001	0.14 (0.08-0.24)	<.001
Tertile 1 vs tertile 3	54.6 (33.2-90.0)	<.001	52.5 (31.7-86.7)	<.001

Abbreviations: eGFRdiff_cys-cr_, difference between cystatin C– and creatinine-based estimated glomerular filtration rate; NA, not applicable.

^a^ Demographic-adjusted model: adjusted for age, sex, race or ethnicity, and creatinine-based eGFR.

^b^ Fully adjusted model: adjusted for demographic-adjusted model and diabetes, hypertension, cardiovascular disease, heart failure, amputation, chronic obstructive pulmonary disease, angiotensin-converting enzyme or angiotensin-receptor blocker, steroids, log urine protein-to-creatinine ratio, and waist circumference.

^c^ All covariates are from the baseline examination except creatinine-based eGFR, urine protein-to-creatinine ratio, waist circumference, and eGFRdiff_cys-cr_, which were time-updated.

^d^ Within-participant slopes were estimated from a joint model of eGFR trajectory and survival or dropout. In slope models, all covariates were from baseline examination except creatinine-based eGFR, urine protein-to-creatinine ratio, and waist circumference, which were time-updated.

### Secondary Analyses

There was no interaction by age, sex, race, or eGFRcr for ESKD in time-updated subgroup analyses (eTable 3 in the Supplement). The association between eGFRdiff_cys-cr_ and mortality differed by self-reported race when we used the race-free 2021 CKD-EPI eGFRcr equation. However, when we repeated the interaction analyses using the 2009 CKD-EPI eGFRcr equation,^29^ which calibrated for race, there was no interaction. In secondary longitudinal analyses, we found that mean eGFRcr slope was similar across tertiles of eGFRdiff_cys-cr_ slope while mean eGFRcys was substantially more negative in the lowest tertiles of eGFRdiff_cys-cr_ slope and more positive in the highest (eTable 2 in the Supplement). Slopes of eGFRdiff_cys-cr_ yielded additional prognostic information beyond slope of eGFRcr for the outcome of mortality but not for ESKD (eTable 4 in the Supplement).

## Discussion

In this cohort of participants with CKD, we investigated the associations of eGFRdiff_cys-cr_ with ESKD and all-cause mortality using baseline, time-updated, and longitudinal slopes of eGFRdiff_cys-cr_. One-third of participants had baseline eGFRcys and eGFRcr values that differed by more than 15 mL/min/1.73 m^2^. Compared with participants in whom eGFRcys and eGFRcr were similar, those with a negative eGFRdiff_cys-cr_ (eGFRcys lower than eGFRcr) had higher risk of ESKD and mortality; those with positive eGFRdiff_cys-cr_ (eGFRcys higher than eGFRcr) had lower risk of these outcomes. Time-updated measures of eGFRdiff_cys-cr_ yielded additional prognostic information beyond baseline eGFRdiff_cys-cr._ Furthermore, in longitudinal analyses, participants whose eGFRcys declined more quickly than eGFRcr had an 8.2-fold higher risk of mortality compared with participants whose eGFRdiff_cys-cr_ remained unchanged over time. These associations were independent of baseline eGFRdiff_cys-cr_. Taken together, our study suggests that among persons with large differences between eGFRcys and eGFRcr, the prognoses for both ESKD and mortality are significantly associated with whether eGFRcys was lower or higher than eGFRcr. Our longitudinal findings further suggest that repeating both serum creatinine and cystatin C annually provides substantially more information about an individual’s evolving health status than only assessing cystatin C at a single time point, as current guidelines suggest.^17^

To our knowledge, this is the first study to examine the association between eGFRdiff_cys-cr_ and ESKD. Prior studies have shown that eGFRcys can improve ESKD prognostication^31,32^; however, these analyses compared associations of eGFRcys and eGFRcr with outcomes at the population level. Given the recent NKF and ASN recommendations,^7,15^ cystatin C is likely to become more widely used in clinical practice, and it will become increasingly apparent that many individual patients have 2 widely disparate eGFR values.^5,6^ Recently, members of our research team investigated the difference between eGFRcys and eGFRcr as a prognostic indicator. In analyses from the Systolic Blood Pressure Intervention Trial and Cardiovascular Health Study cohorts, higher baseline eGFRdiff_cys-cr_ was associated with lower risk of all-cause mortality as well as frailty, falls, hospitalizations, and CVD events.^5,6^ However, prior studies have not evaluated kidney-specific end points among persons with CKD, a population for whom accurate GFR estimation and risk stratification are essential. Hence, our findings that eGFRdiff_cys-cr_ was associated with risk for ESKD and mortality could inform how these 2 markers might be best used in clinical practice.

The second major finding of this study is that changes in the difference between eGFRcys and eGFRcr over time, represented by time-updated eGFRdiff_cys-cr_ and slope of eGFRdiff_cys-cr_, yielded significant prognostic information beyond the baseline eGFRdiff_cys-cr_. The valuable prognostic information provided by time-updated eGFRdiff_cys-cr_ suggests that there were important fluctuations in eGFRcys or eGFRcr, perhaps driven by changes in health status, that warrant repeated measurements of both cystatin C and creatinine. Declining health status often leads to decreased physical activity, sarcopenia, and worsening nutrition, all of which reduce creatinine production and diminish its performance as a GFR marker.^10,11,12,13^ In this scenario for patients with declining kidney function, eGFRcr may remain deceptively stable or even increase, whereas eGFRcys would be unaffected by these nonrenal factors. Consequently, eGFRdiff_cys-cr_ would become progressively negative as overall health status worsens and kidney function declines, which would explain the higher risk for adverse outcomes. Our longitudinal slope findings further illustrated this concept by revealing that participants who had eGFRcr declining more slowly than eGFRcys (first tertile of eGFRdiff_cys-cr_ slope) had substantially higher risk of mortality than participants in whom eGFRcys and eGFRcr declined in parallel (second tertile of eGFRdiff_cys-cr_ slope). Notably, despite the drastic differences in mortality risks across tertiles of eGFRdiff_cys-cr_ slope, mean eGFRcr slopes were similar. The lack of association between slope of eGFRdiff_cys-cr_ and ESKD in fully adjusted models is likely attributable to the association between eGFRcr slope and ESKD and our adjustment for eGFRcr in the eGFRdiff_cys-cr_ models. Furthermore, ESKD is a somewhat biased outcome, particularly in the context of our slope analyses, since clinicians rely heavily on eGFRcr and the rate of eGFRcr decline to determine timing of dialysis initiation or transplant referral. Nevertheless, eGFRdiff_cys-cr_ slope was associated with mortality, and the direction of its associated risk with ESKD was consistent. Furthermore, we found that unadjusted risks for both ESKD and mortality varied based on the annual change in eGFRdiff_cys-cr_ across the distribution of baseline eGFRdiff_cys-cr_. Collectively, these findings highlight the prognostic relevance of assessing both eGFRcys and eGFRcr longitudinally rather than relying on a single eGFRcys measure.

Our findings suggest that monitoring of eGFRcys is critical for patients with the worst health status, as they are the most likely to have their eGFRcr biased by creatinine underproduction and thus to have more negative eGFRdiff_cys-cr_. Conversely, participants with positive eGFRdiff_cys-cr_ or steeper declines in eGFRcr than eGFRcys (positive eGFRdiff_cys-cr_ slope) were relatively healthy and had lower risks for ESKD and mortality. In these individuals, higher levels of physical activity and muscle mass may increase creatinine production, leading to underestimation of kidney function and overestimation of ESKD and mortality risk. Hypothetically, accounting for all non-GFR factors that may possibly affect creatinine more than cystatin C over time could explain the substantial differences in risk of adverse outcomes among patients with varying degrees of eGFRdiff_cys-cr_. However, eGFRdiff_cys-cr_ captures a conglomerate of non-GFR factors that are associated with overall health status. Accurately measuring and quantifying all of these factors is both impractical and likely impossible in either well-designed research cohorts or in real-world clinical practice.^14^ Our findings show that obtaining a cystatin C measurement and assessing differences between eGFRcr and eGFRcys is a simple approach to distinguish risk among individuals with CKD.

While non-GFR factors associated with cystatin C also exist, such as obesity, steroid use, and possibly inflammation, their associations with serum cystatin C levels are smaller in magnitude than those of non-GFR factors associated with serum creatinine.^16,33,34,35^ We adjusted for waist circumference and steroid use in our baseline, time-updated, and slope analyses. Our results remained robust after additional adjustment for CRP in exploratory analyses. These results reinforce prior studies demonstrating that adjustment for markers of inflammation does not affect the prognostic value of cystatin C.^36,37,38^

Our findings have important clinical implications. Persons with large negative or positive eGFRdiff_cys-cr_ comprise high- and low-risk CKD subgroups for whom creatinine alone does not adequately capture ESKD and mortality risk. Recently, the NKF and ASN recommended increased use of the combined eGFR equation, which incorporates both cystatin C and creatinine to balance the influence of their non-GFR contributing factors.^7,15^ However, whether this combined eGFR equation should be applied among individuals in whom eGFRcys and eGFRcr are discrepant by more than 15 mL/min/1.73 m^2^ is unclear.^39^ Our findings indicate that a large eGFRdiff_cys-cr_ should prompt careful consideration as to whether creatinine may be heavily biased by factors unrelated to GFR, such as poor health status and sarcopenia. Use of the combined eGFR equation under these circumstances may misrepresent kidney function and prognosis. Recently, only 7% of surveyed laboratories in the US offered cystatin C tests.^40^ The higher cost of reagents for cystatin C compared with that for creatinine (approximately $4 vs $0.20 per test) is a commonly cited barrier to more widespread implementation.^39^ We anticipate that this cost barrier may be overcome with increased demand for cystatin C in clinical practice.

### Limitations

There are some important limitations of our study. First, previous studies have found potential associations of cystatin C with thyroid dysfunction,^41,42^ but we did not have data on thyroid-related diagnoses or medications. Second, we encourage caution in applying our findings to all racial or ethnic groups, as most participants in our study had self-identified as non-Hispanic Black or White. Future research must extend these analyses to other racial and ethnic groups. Third, because entry criteria into the CRIC Study were based on eGFRcr values, the range of eGFRcr, but not of eGFRcys, was restricted to between 20 and 70 mL/min/1.73 m^2^. This truncation of the eGFRcr distribution by study design may have resulted in the exclusion of participants who would have been categorized in the negative or positive eGFRdiff_cys-cr_ groups. Additionally, we cannot rule out residual confounding due to the observational study design.

## Conclusions

This cohort study found that substantial differences between eGFRcys and eGFRcr were common and that eGFRdiff_cys-cr_ conveys important prognostic information that is currently missed when only eGFRcr is monitored over time. When eGFRcys and eGFRcr were discrepant by more than 15 mL/min/1.73 m^2^, risk of ESKD and mortality was associated with whether eGFRcys was lower or higher than eGFRcr. The widening of eGFRdiff_cys-cr_ over time, which depicted divergence between the slopes of eGFRcys and eGFRcr, captured clinically important changes in health status that manifested as significant associations with mortality risk. Our results support the clinical utility in regularly obtaining measures of cystatin C in addition to creatinine throughout the longitudinal care of persons with CKD.

## Notes

Supplement.
eMethods.
**eTable 1.** Associations of eGFRdiff_cys-cr_ With End-stage Kidney Disease (ESKD) and All-Cause Mortality in Exploratory Analyses Adding Adjustment for Serum Albumin, Hemoglobin, and C-Reactive Protein **eTable 2.** Clinical Events and Slopes of eGFRcys and eGFRcr by Tertile of eGFRdiff_cys-cr_ **eTable 3.** Multivariable Adjusted Associations of eGFRdiff_cys-cr_ With End-stage Kidney Disease (ESKD) and All-Cause Mortality, Stratified by Subgroup **eTable 4.** Individually-Adjusted Associations of Slopes of eGFRdiff_cys-cr_, eGFRcr, and eGFRcys With End-stage Kidney Disease (ESKD) and All-Cause Mortality **eFigure 1.** Distribution of Baseline eGFRdiff_cys-cr_ **eFigure 2.** Scatterplot of eGFRcr vs eGFRdiff_cys-cr_ at Baseline
