# Supplementary material for: Association of Intraindividual Difference in Estimated Glomerular Filtration Rate by Creatinine vs Cystatin C and End-stage Kidney Disease and Mortality
Source: JAMA Netw Open. 2022 Feb 17;5(2):e2148940. doi: 10.1001/jamanetworkopen.2021.48940 (PMC8855239; doi:10.1001/jamanetworkopen.2021.48940)
Supplement: Supplement. — eMethods. eTable 1. Associations of eGFRdiffcys-cr With End-stage Kidney Disease (ESKD) and All-Cause Mortality in Exploratory Analyses Adding Adjustment for Serum Albumin, Hemoglobin, and C-Reactive Protein eTable 2. Clinical Events and Slopes of eGFRcys and eGFRcr by Tertile of eGFRdiffcys-cr eTable 3. Multivariable Adjusted Associations of eGFRdiffcys-cr With End-stage Kidney Disease (ESKD) and All-Cause Mortality, Stratified by Subgroup eTable 4. Individually-Adjusted Associations of Slopes of eGFRdiffcys-cr, eGFRcr, and eGFRcys With End-stage Kidney Disease (ESKD) and All-Cause Mortality eFigure 1. Distribution of Baseline eGFRdiffcys-cr eFigure 2. Scatterplot of eGFRcr vs eGFRdiffcys-cr at Baseline [file jamanetwopen-e2148940-s001.pdf]

Supplemental Online Content

Chen DC, Shlipak MG, Scherzer R, et al. Association of intraindividual difference in estimated glomerular filtration rate by creatinine vs cystatin C and end-stage kidney disease and mortality. *JAMA Netw Open*. 2022;5(2):e2148940. doi:10.1001/jamanetworkopen.2021.48940

eMethods.
eTable 1. Associations of eGFRdiff$_{cys-cr}$ With End-stage Kidney Disease (ESKD) and All-Cause Mortality in Exploratory Analyses Adding Adjustment for Serum Albumin, Hemoglobin, and C-Reactive Protein
eTable 2. Clinical Events and Slopes of eGFRcys and eGFRcr by Tertile of eGFRdiff$_{cys-cr}$
eTable 3. Multivariable Adjusted Associations of eGFRdiff$_{cys-cr}$ With End-stage Kidney Disease (ESKD) and All-Cause Mortality, Stratified by Subgroup
eTable 4. Individually-Adjusted Associations of Slopes of eGFRdiff$_{cys-cr}$, eGFRcr, and eGFRcys With End-stage Kidney Disease (ESKD) and All-Cause Mortality
eFigure 1. Distribution of Baseline eGFRdiff$_{cys-cr}$
eFigure 2. Scatterplot of eGFRcr vs eGFRdiff$_{cys-cr}$ at Baseline

This supplemental material has been provided by the authors to give readers additional information about their work.

© 2022 Chen DC et al. *JAMA Network Open.*

eMethods.

Independent variables

Serum cystatin C levels were measured using particle-enhanced immunonephelometric assay on the Siemens BN II System (Siemens Health Care Diagnostics, Inc., Tarrytown, NY) with an inter-assay coefficient of variation (CV) of 4.9%.[1] Cystatin C was internally standardized to account for assay drift over time. Serum creatinine was measured using an enzyme-based assay on the Hitachi Vitros 950 VT (Ortho-Clinical Diagnostics, Inc. Rochester, NY), with a CV of 1.1%, and calibration traceable to an isotope dilution mass spectrometry reference measurement procedure.[1, 2]

Joint models

We used a joint model to simultaneously evaluate repeated measures of $eGFRdiff_{cys\text{-}cr}$ and time-to-event data.[3] This approach reduces bias, accounts for informative censoring, and improves precision relative to more traditional survival analyses.[4-8] Our joint model linked two sub-models: a linear mixed effect model assessing changes in $eGFRdiff_{cys\text{-}cr}$ values across the baseline and first three annual study visits; and an exponential survival model beginning at the baseline study visit. The linear mixed effect sub-model assumed random effects for both slope and intercept. Through the joint models, we obtained within-subject estimates of $eGFRdiff_{cys\text{-}cr}$ slope and intercept. We created tertiles of $eGFRdiff_{cys\text{-}cr}$ slope and evaluated the association between these tertiles and ESKD and mortality in Fine-Gray and Cox models. In these time-to-event models of $eGFRdiff_{cys\text{-}cr}$ slope, we adjusted for time-updated eGFRcr, UPCR, and waist circumference.

Model diagnostics

Examination of variance inflation factor and condition index diagnostics found no evidence of collinearity between covariates in our models. Proportional hazards assumptions were tested using Schoenfeld residuals. We identified non-proportional hazards in our model evaluating the association between slope of $eGFRdiff_{cys\text{-}cr}$ and mortality. Compared to tertile 2 of $eGFRdiff_{cys\text{-}cr}$ slope, tertile 1 was associated with increasing mortality risk while tertile 3 was associated with decreasing mortality risk over time, with overall unadjusted hazard ratios of 5.84 (95% CI: 5.06, 6.73) and 0.13 (95% CI: 0.09, 0.19), respectively. These overall hazard ratios represent the weighted average of the true hazard ratios over the entire follow-up period.[9]

© 2022 Chen DC et al. *JAMA Network Open.*

Handling of missing variables

Baseline variables with <2% missing data included serum albumin, serum hemoglobin, steroid use, waist circumference, HTN, ACEI/ARB use, and COPD; UPCR had 5% missing, and amputation and CRP had 27% missing. Multiple imputation with the Markov chain Monte Carlo method for arbitrary missing multivariate normal data was used to impute missing covariates, with 20 imputations to ensure ~95% relative efficiency.

Ethical review of study

This current study was determined to be exempt from review by the University of California, San Francisco IRB, as all data were de-identified. Data were obtained from the National Institute of Diabetes and Digestive and Kidney Diseases (NIDDK) repository in April 2021.

© 2022 Chen DC et al. *JAMA Network Open.*

eMethods References

1. Anderson AH, Yang W, Hsu CY, et al. Estimating GFR among participants in the Chronic Renal Insufficiency Cohort (CRIC) Study. *Am J Kidney Dis*. Aug 2012;60(2):250-61. doi:10.1053/j.ajkd.2012.04.012
2. Joffe M, Hsu CY, Feldman HI, Weir M, Landis JR, Hamm LL. Variability of creatinine measurements in clinical laboratories: results from the CRIC study. *Am J Nephrol*. 2010;31(5):426-34. doi:10.1159/000296250
3. Garcia-Hernandez A, Rizopoulos D. %JM: A SAS Macro to Fit Jointly Generalized Mixed Models for Longitudinal Data and Time-to-Event Responses. SAS macro; joint modeling; time-to-event; longitudinal; generalized mixed models; shared parameter models; survival data. *2018*. 2018-04-28 2018;84(12):29. doi:10.18637/jss.v084.i12
4. Wulfsohn MS, Tsiatis AA. A joint model for survival and longitudinal data measured with error. *Biometrics*. Mar 1997;53(1):330-9.
5. Pauler DK, Finkelstein DM. Predicting time to prostate cancer recurrence based on joint models for non-linear longitudinal biomarkers and event time outcomes. *Stat Med*. Dec 30 2002;21(24):3897-911. doi:10.1002/sim.1392
6. Henderson R, Diggle P, Dobson A. Joint modelling of longitudinal measurements and event time data. *Biostatistics*. Dec 2000;1(4):465-80. doi:10.1093/biostatistics/1.4.465
7. Chesnaye NC, Tripepi G, Dekker FW, Zoccali C, Zwinderman AH, Jager KJ. An introduction to joint models-applications in nephrology. *Clin Kidney J*. 2020;13(2):143-149. doi:10.1093/ckj/sfaa024
8. Rizopoulos D. Dynamic predictions and prospective accuracy in joint models for longitudinal and time-to-event data. *Biometrics*. Sep 2011;67(3):819-29. doi:10.1111/j.1541-0420.2010.01546.x
9. Stensrud MJ, Hernán MA. Why Test for Proportional Hazards? *JAMA*. 2020;323(14):1401-1402. doi:10.1001/jama.2020.1267

© 2022 Chen DC et al. *JAMA Network Open.*

eTable 1. Associations of $eGFRdiff_{cys\text{-}cr}$ With End-stage Kidney Disease (ESKD) and All-Cause Mortality in Exploratory Analyses Adding Adjustment for Serum Albumin, Hemoglobin, and C-Reactive Protein

	Subhazard Ratio (95% CI) for ESKD		Hazard Ratio (95% CI) for All-Cause Mortality	
	**Fully-adjusted Model[c]**	**Exploratory Model[d]**	**Fully-adjusted Model[c]**	**Exploratory Model[d]**
**Baseline measures**<br>Categorical $eGFRdiff_{cys - cr}$, mL/min/1.73 m^2				
< -15	1.00 (0.65, 1.52), p=0.99	0.89 (0.57, 1.37), p=0.59	1.86 (1.40, 2.48), p<0.0001	1.76 (1.32, 2.34), p=0.0001
-15 to 15	Ref	Ref	Ref	Ref
≥ 15	0.73 (0.59, 0.89), p=0.0021	0.73 (0.60, 0.90), p=0.0026	0.68 (0.58, 0.81), p<0.0001	0.72 (0.61, 0.84), p<0.0001
**Time-updated measures[a]**<br>Categorical $eGFRdiff_{cys - cr}$, mL/min/1.73 m^2				
< -15	1.83 (1.10, 3.04), p=0.020	1.76 (1.06, 2.94), p=0.029	3.03 (2.19, 4.19), p<0.0001	2.91 (2.09, 4.04), p<0.0001
-15 to 15	Ref	Ref	Ref	Ref
≥ 15	0.50 (0.35, 0.71), p=0.0001	0.50 (0.35, 0.72), p=0.0002	0.58 (0.45, 0.75), p<0.0001	0.62 (0.48, 0.79), p=0.0002
**Slope of eGFRdiff[b]**,<br>mL/min/1.73 m^2/year				
Tertile 1, -7.4 to -0.5	1.19 (0.98, 1.46), p=0.082	1.20 (0.98, 1.45), p=0.072	8.20 (6.37, 10.56), p<0.0001	8.14 (6.33, 10.49), p<0.0001
Tertile 2, -0.5 to + 0.2	Ref	Ref	Ref	Ref
Tertile 3, +0.2 to + 25.6	0.86 (0.70, 1.06), p=0.16	0.87 (0.70, 1.07), p=0.18	0.14 (0.08, 0.24), p<0.0001	0.14 (0.08, 0.24), p<0.0001

[a] All covariates are from the baseline exam except creatinine-based estimated glomerular filtration rate (eGFRcr), urine protein-to-creatinine ratio (UPCR), waist circumference, and $eGFRdiff_{cys - cr}$, which were time-updated
[b] Within-subject slopes estimated from joint model of eGFR trajectory and survival or dropout, adjusted for baseline $eGFRdiff_{cys - cr}$. In slope models, all covariates were from baseline exam except creatinine-based estimated glomerular filtration rate (eGFRcr), urine protein-to-creatinine ratio (UPCR), waist circumference, which were time-updated.

[c] **Fully-adjusted model** adjusted for age, sex, race or ethnicity, and eGFRcr, diabetes, hypertension, cardiovascular disease, heart failure, amputation, chronic obstructive pulmonary disease, angiotensin-converting enzyme or angiotensin-receptor blocker, steroids, log(UPCR), and waist circumference
[d] **Exploratory model** adjusted for covariates in the fully-adjusted model and serum albumin, hemoglobin, and log(C-reactive protein)

Subhazard ratios for ESKD were obtained using Fine and Gray proportional subhazards regression, modeling mortality as a competing risk. Hazard ratios for mortality were obtained using Cox proportional hazards models.

© 2022 Chen DC et al. *JAMA Network Open.*

eTable 2. Clinical Events and Slopes of eGFRcys and eGFRcr by Tertile of $eGFRdiff_{cys\text{-}cr}$

Tertile of $eGFRdiff_{cys\text{-}cr}$ slope	Tertile 1	Tertile 2	Tertile 3	Total
Number of participants	1652	1652	1652	4,956
ESKD, n (% of tertile)	534 (32)	385 (23)	254 (15)	1,173
Death, n (% of tertile)	1,020 (62)	245 (15)	33 (2)	1,298
Death after ESKD event, n (% of tertile)	452 (27)	77 (5)	4 (0.2)	533
$eGFRdiff_{cys\text{-}cr}$ slope, mL/min/1.73 m^2/year	-7.9 to -0.83	-0.83 to -0.06	-0.06 to 24.2	
eGFRcys slope, mean (SD), mL/min/1.73 m^2/year	-3.77 ± 1.90	-2.08 ± 1.77	+0.42 ± 2.66	
eGFRcr slope, mean (SD), mL/min/1.73 m^2/year	-2.19 ± 2.65	-1.84 ± 2.25	-1.41 ± 2.14	

Within-subject slopes of $eGFRdiff_{cys - cr}$, eGFRcys, and eGFRcr were estimated from joint models of the trajectory of each eGFR value and survival

© 2022 Chen DC et al. *JAMA Network Open.*

eTable 3. Multivariable Adjusted Associations of $eGFRdiff_{cys-cr}$ With End-stage Kidney Disease (ESKD) and All-Cause Mortality, Stratified by Subgroup

	**HR (95% CI) for ESKD**		**HR (95% CI) for All-Cause Mortality**	
	**Age < 60**	**Age ≥ 60**	**Age < 60**	**Age ≥ 60**
**Baseline eGFRdiff** *, mL/min/1.73 m^2				
< -15	1.03 (0.60, 1.76), p=0.91	0.90 (0.46, 1.77), p=0.76	1.92 (1.18, 3.11), p=0.0081	1.83 (1.31, 2.57), p=0.0004
-15 to 15	Ref	Ref	Ref	Ref
≥ 15	0.72 (0.56, 0.91), p=0.0074	0.75 (0.55, 1.04), p=0.086	0.73 (0.56, 0.95), p=0.020	0.66 (0.54, 0.80), p<0.0001
Age x eGFRdiff interaction	p=0.91		p=0.80	
	**HR (95% CI) for ESKD**		**HR (95% CI) for All-Cause Mortality**	
	**Female**	**Male**	**Female**	**Male**
**Baseline eGFRdiff** *, mL/min/1.73 m^2				
< -15	1.37 (0.76, 2.47), p=0.30	0.83 (0.48, 1.44), p=0.51	1.91 (1.19, 3.07), p=0.0074	1.83 (1.30, 2.58), p=0.0005
-15 to 15	Ref	Ref	Ref	Ref
≥ 15	0.63 (0.45, 0.87), p=0.0047	0.79 (0.62, 1.02), p=0.072	0.57 (0.43, 0.75), p<0.0001	0.75 (0.62, 0.91), p=0.0041
Sex x eGFRdiff interaction	p=0.21		p=0.25	
	**HR (95% CI) for ESKD**		**HR (95% CI) for All-Cause Mortality**	
	**Black**	**Non-Black**	**Black**	**Non-Black**
**Baseline eGFRdiff** *, mL/min/1.73 m^2				
< -15	0.85 (0.40, 1.79), p=0.67	1.03 (0.61, 1.73), p=0.92	1.45 (0.86, 2.43), p=0.16	2.00 (1.44, 2.79), p<0.0001
-15 to 15	Ref	Ref	Ref	Ref
≥ 15	0.81 (0.64, 1.02), p=0.073	0.59 (0.41, 0.85), p=0.0044	0.78 (0.64, 0.96), p=0.017	0.54 (0.41, 0.71), p<0.0001
Race x eGFRdiff interaction	p=0.29		p=0.042	
	**HR (95% CI) for ESKD**		**HR (95% CI) for All-Cause Mortality**	
	**eGFRcr < 45**	**eGFRcr ≥ 45**	**eGFRcr < 45**	**eGFRcr ≥ 45**
**Baseline eGFRdiff** *, mL/min/1.73 m^2				
< -15	0.94 (0.39, 2.25), p=0.89	0.97 (0.62, 1.52), p=0.89	1.53 (0.76, 3.07), p=0.24	2.04 (1.49, 2.79), p<0.0001
-15 to 15	Ref	Ref	Ref	Ref
≥ 15	0.73 (0.56, 0.95), p=0.020	0.72 (0.54, 0.96), p=0.027	0.61 (0.48, 0.77), p<0.0001	0.77 (0.61, 0.97), p=0.025
eGFRcr x eGFRdiff interaction	p=0.99		p=0.29	

Fully-adjusted model adjusted for age, sex, race or ethnicity, and eGFRcr, diabetes, hypertension, cardiovascular disease, heart failure, amputation, chronic obstructive pulmonary disease, angiotensin-converting enzyme or angiotensin-receptor blocker, steroids, log(urine protein-to-creatinine ratio), and waist circumference

© 2022 Chen DC et al. *JAMA Network Open.*

eTable 4. Individually-Adjusted Associations of Slopes of $eGFRdiff_{cys\text{-}cr}$, eGFRcr, and eGFRcys With End-stage Kidney Disease (ESKD) and All-Cause Mortality

	**Subhazard Ratio (95% CI) for ESKD**		**Hazard Ratio (95% CI) for All-Cause Mortality**	
	**Demographic-adjusted [b]**	**Fully-adjusted [c]**	**Demographic-adjusted [b]**	**Fully-adjusted [c]**
**Slope of $eGFRdiff_{cys\text{-}cr}$,** per mL/min/1.73 m^2/year [a]				
**Tertile 1**	1.50 (1.31, 1.72), p<0.0001	1.43 (1.22, 1.68), p<0.0001	5.13 (4.38, 6.00), p<0.0001	4.54 (3.87, 5.32), p<0.0001
**Tertile 2**	Ref	Ref	Ref	Ref
**Tertile 3**	0.65 (0.56, 0.76), p<0.0001	0.91 (0.77, 1.09), p=0.31	0.11 (0.08, 0.16), p<0.0001	0.11 (0.08, 0.16), p<0.0001
**Slope of eGFRcr,** per mL/min/1.73 m^2/year [a]				
**Tertile 1**	3.59 (3.12, 4.14), p<0.0001	2.26 (1.93, 2.64), p<0.0001	2.60 (2.29, 2.95), p<0.0001	2.17 (1.90, 2.49), p<0.0001
**Tertile 2**	Ref	Ref	Ref	Ref
**Tertile 3**	0.56 (0.46, 0.70), p<0.0001	0.72 (0.58, 0.90), p=0.0038	0.61 (0.51, 0.74), p<0.0001	0.64 (0.53, 0.77), p<0.0001
**Slope of eGFRcys,** per mL/min/1.73 m^2/year [a]				
**Tertile 1**	2.74 (2.39, 3.14), p<0.0001	1.86 (1.61, 2.15), p<0.0001	4.59 (3.98, 5.28), p<0.0001	4.25 (3.68, 4.91), p<0.0001
**Tertile 2**	Ref	Ref	Ref	Ref
**Tertile 3**	0.52 (0.41, 0.65), p<0.0001	0.70 (0.55, 0.88), p=0.0029	0.43 (0.33, 0.56), p<0.0001	0.43 (0.33, 0.56), p<0.0001

[a] Within-subject slopes estimated from joint model of $eGFRdiff_{cys\text{-}cr}$, eGFRcr, or eGFRcys trajectory and survival or dropout. All covariates in slope analyses were from baseline exam.

[b] **Demographic-adjusted model:** adjusted for age, sex, and race or ethnicity
[c] **Fully-adjusted model:** adjusted for demographic-adjusted model and diabetes, hypertension, cardiovascular disease, heart failure, amputation, chronic obstructive pulmonary disease, angiotensin-converting enzyme or angiotensin-receptor blocker, steroids, log(urine protein-to-creatinine ratio), and waist circumference

Subhazard ratios for ESKD were obtained using Fine and Gray proportional subhazards regression, modeling mortality as a competing risk. Hazard ratios for mortality were obtained using Cox proportional hazards models.

© 2022 Chen DC et al. *JAMA Network Open.*

eFigure 1. Distribution of Baseline eGFRdiff$_{cys\text{-}cr}$

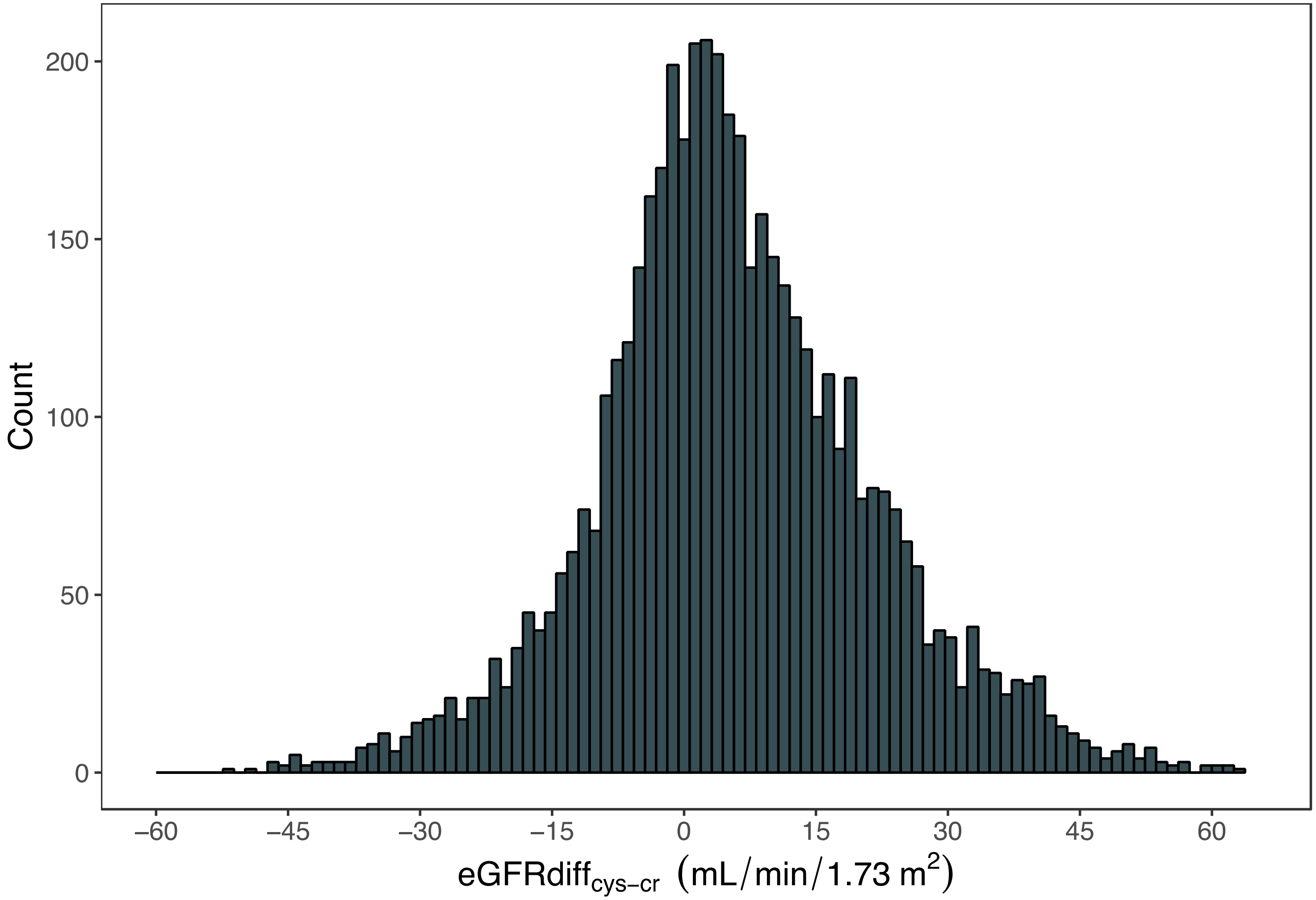

Abbreviation: eGFRdiff$_{cys-cr}$, estimated glomerular filtration rate difference

© 2022 Chen DC et al. *JAMA Network Open.*

eFigure 2. Scatterplot of eGFRcr vs $eGFRdiff_{cys\text{-}cr}$ at Baseline

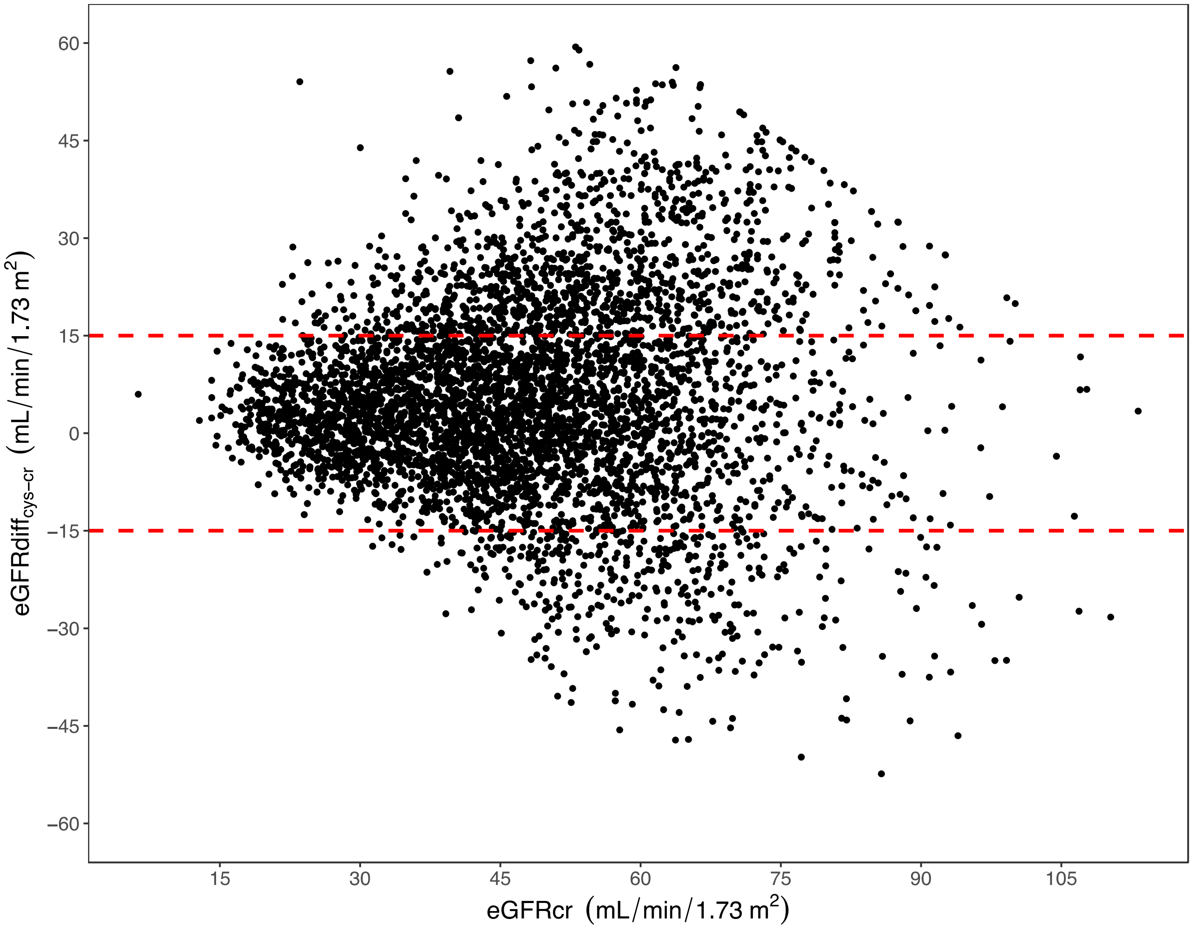

Abbreviation: $eGFRdiff_{cys - cr}$, estimated glomerular filtration rate difference; eGFRcr, creatinine-based estimated glomerular filtration rate based on the 2021 CKD Epidemiology Collaboration (CKD-EPI) equation

© 2022 Chen DC et al. *JAMA Network Open.*
